# Evolutionary approach to construct robust codes for DNA-based data storage

**DOI:** 10.3389/fgene.2023.1158337

**Published:** 2023-03-20

**Authors:** Abdur Rasool, Qingshan Jiang, Yang Wang, Xiaoluo Huang, Qiang Qu, Junbiao Dai

**Affiliations:** ^1^ Shenzhen Institute of Advanced Technology, Chinese Academy of Sciences, Shenzhen, China; ^2^ Shenzhen College of Advanced Technology, University of Chinese Academy of Sciences, Beijing, China

**Keywords:** biocomputing, DNA coding sets, bioconstrained codes, MFO, DNA data storage

## Abstract

DNA is a practical storage medium with high density, durability, and capacity to accommodate exponentially growing data volumes. A DNA sequence structure is a biocomputing problem that requires satisfying bioconstraints to design robust sequences. Existing evolutionary approaches to DNA sequences result in errors during the encoding process that reduces the lower bounds of DNA coding sets used for molecular hybridization. Additionally, the disordered DNA strand forms a secondary structure, which is susceptible to errors during decoding. This paper proposes a computational evolutionary approach based on a synergistic moth-flame optimizer by Levy flight and opposition-based learning mutation strategies to optimize these problems by constructing reverse-complement constraints. The MFOS aims to attain optimal global solutions with robust convergence and balanced search capabilities to improve DNA code lower bounds and coding rates for DNA storage. The ability of the MFOS to construct DNA coding sets is demonstrated through various experiments that use 19 state-of-the-art functions. Compared with the existing studies, the proposed approach with three different bioconstraints substantially improves the lower bounds of the DNA codes by 12–28% and significantly reduces errors.

## 1 Introduction

The natural objective of DNA-encoding methods is to provide high-density, error-free, and stable DNA codes by dynamic programming (Yim et al., 2014; Erlich and Zielinski, 2017; Organick et al., 2018; Lu et al., 2023). However, the computational capacity of dynamic programming remains infeasible with big digital data and biological sequences. Consequently, substantial efforts have been invested in finding efficient heuristic/optimization algorithms to tackle this feasibility (Cao et al., 2020a). These algorithms produce upper and lower bounds in DNA sequence data by dynamic programming. A sequence $a_{n}\geq k$ , if all its terms are greater than or equal to a number, k , is called the lower bound of the sequence, while a sequence $a_{n}\leq k^{\prime }$ , if all its terms are less than or equal to a number, $k^{\prime }$ , is called the upper bound of the sequence (Chee and Ling, 2008). The accuracy of both the upper and lower bounds is measured concurrently by comparing them. No matter how strong an upper bound is, comparing it to a weak lower bound does not prove that it is close to the optimum. Therefore, it is essential to make lower-bound improvements. However, let us ask a more modest question. How can the improvement in the lower bounds of DNA sequence data enable the achievement of the objectives of DNA-encoding methods? A precise answer negates the accomplishment of all objectives except the stable DNA codes by reducing the non-specific hybridization errors. However, the task of constructing robust DNA-coding sets is still challenging due to sequence errors. Molecular-based computation delivers a set of sequences with the corrupt version of DNA codes, including symbol deletion or substitution and high-magnitude errors in the code words, which lose the sequences during the hybridization process (Yim et al., 2014; Li et al., 2021a). These massive errors decrease the lower bounds between the different numbers of sequences. Consequently, the DNA synthesis process becomes noisy. It is more expensive to synthesize and relatively reduces the stability of DNA data storage, which can be biotechnologically quantified, controlled, and engineered (Song et al., 2021; Song et al., 2022).

Biotechnology and bioengineering recently played a vital role in DNA computation as nanobiotechnology synthesizes DNA. Research communities are still combating errors in DNA-coding sets with different computational methods (Grass et al., 2015; Yazdi et al., 2017; Deng et al., 2019). One of the computational methods is evolutionary computation, which comprises a set of biologically inspired algorithms. For example, the author showed the chaos whale optimization with sine and cosine functions to construct the DNA sequence set (Li et al., 2021b). Similarly, Xiaoru and Ling (2021) delivered an evolutionary-based equilibrium optimization with a random search model to overcome the error rate in DNA-coding sets. These studies contributed significantly to advancing evolutionary approaches in DNA storage. However, the results still lacked an improvement in the diversity of the sampled population.

For this purpose, the effectiveness of the optimization algorithm will have to increase by designing the mutation strategies. Wang et al. (2020) considered the differential evaluation algorithm with a two-level local search strategy to improve convergence. The general purpose of such strategies is to avoid the local minima and prevent the population from being similar. The improvement in evolutionary algorithms by mutation strategies also significantly impacts the improvement of the lower bounds of DNA-coding sets. A multi-verse optimizer (MVO) was made synergistic by damping the strategy to gain a stable state in global search, and it also reported 4–16% of improved DNA-coding sets (Cao et al., 2020b). Rasool et al. (2022a) used the opposition-based learning (OBL) mutation strategy (Dong et al., 2017) for three-dimensional search space with a meta-heuristic algorithm (Mirjalili, 2015) to generate the DNA-coding sets for data storage. The codes generated by the improved algorithm (MFOL) enhanced the DNA-coding sets over the altruistic algorithm (Limbachiya et al., 2018).

Nevertheless, these evolutionary studies have performed well by enhancing the efficiency of optimization algorithms by the mutation strategies, but they focus on particular biological coding constraints. The satisfaction of these constraints controls the oligonucleotide errors in the DNA sequences (Sager and Stefanovic, 2006; Song et al., 2018; Li et al., 2021b; Cao et al., 2022). For instance, the GC-content and Hamming distance constraints were considered for the oligonucleotide libraries construction by reducing the hybridization errors (Aboluion et al., 2012; Organick et al., 2018; Yang et al., 2020; Rasool et al., 2022b). However, the current approaches and algorithms of DNA code construction by the lower-bound computation do not satisfy the reverse-complement (RC) constraints. However, unsatisfactory reverse-complement constraints are relatively crucial in DNA codes with a high rate of errors, which cause the DNA stability for storage (Sager and Stefanovic, 2006; Yin et al., 2010).

In short, the conventional optimization algorithms have evolved for lower-bound improvements. Still, various functions’ convergence abilities have declined, which was improved by different mutation strategies with different purposes. However, the lower bounds of DNA-coding sets were insufficient for robust DNA storage due to non-specific hybridization errors (Cao et al., 2020b). Although these errors have been tackled by a few biological coding constraints, the extent of error avoidance was low because DNA sequences overlapped, creating non-specific hybridization that formed the secondary structure. DNA sequences with such a structure make the chemical reaction unstable and inactivate DNA synthesis and sequencing (Song et al., 2021). Thus, the coding sets of DNA storage are still facing the lowest global optimal performance with different evolutionary algorithms and mutation strategies to store more information in a shorter sequence with a minimum error.

Thus, in this paper, a bioengineering-based evolutionary approach has been proposed to solve the aforementioned problems. This approach integrates the MFO (Mirjalili, 2015) with two customized mutation strategies (MFOS) and practically applies it to construct DNA-coding sets. We customized the original mutation strategies for compatibility with the optimization algorithms to improve the lower bounds for the DNA code construction. Then, as the main objective of this study, MFOS is used with combinatorial constraints (GC-content, Hamming distance, no-runlength, and RC constraints) to construct DNA-coding sets. The computational RC constraint significantly increases the number of DNA strands (codes) and avoids further non-specific hybridization. The experiments are performed on 19 benchmark functions, and the Wilcoxon rank-sum test is used to evaluate the improved algorithm’s quality. The performance is compared with six existing evolutionary algorithms. The overall contribution of the proposed approach produced DNA-coding sets with improved lower bounds and coding rates compared to previous studies. It is a collaborative effort of information technology and biotechnology domains to solve a bioinformatics-based computational problem for DNA data storage. The following are the noteworthy contributions of this study:

• A novel evolutionary approach is proposed to construct DNA-coding sets by using the MFOS algorithm with two customized mutation strategies for speedy convergence and powerful exploration and exploitation capabilities.
• The novelty of this study is the practical implication of the MFOS algorithm with DNA coding constraints to improve the lower bounds and coding rates of the DNA codes by storing larger data files in the shorter sequence of DNA, and the results are compared with the existing literature.
• Computational RC constraints are satisfied to generate stable DNA codes with our two theorems by eliminating the undesired non-specific hybridization errors due to the secondary structure in DNA sequences. Additionally, an empirical thermodynamic analysis is performed on the RC constraints to validate the DNA coding sets and compare them with the prior constraints.

The rest of this paper is organized as follows. Section 2 briefs the existing DNA coding constraints. Section 3 introduces the evolutionary approach. Section 4 describes the experimental procedure, Section 5 provides result evaluation, and Section 6 reports the conclusion and future work.

## 2 DNA coding constraints

The crucial part of DNA data storage is to construct DNA sequences with minimum errors during the synthesis and sequencing processes. These complicated processes are prone to insertion, substitution, and deletion errors (Yim et al., 2014; Wang et al., 2022). These errors with each nucleotide occur due to the consecutive repetitive subsequences, homopolymers, and GC-content with the minimum and maximum bases. The prior benchmark coding constraints (Cao et al., 2020b), including GC-content, no-runlength (NL), and Hamming distance, have also been used to construct DNA sequences by avoiding those errors. Additionally, this study introduces computation reverse-complement constraints to overcome further non-specific hybridization errors and the secondary structure during the DNA synthesis and sequences with the following mechanism.

A DNA code with length *n* will be a set of codes ( $x_{1}x_{2}x_{3}...x_{n}$ ), while the quaternary alphabets $x_{i}\in \left\{A,C,G,T\right\}$ represent the four DNA nucleotides that form a DNA sequence. For a DNA sequence $x=x_{1}x_{2}x_{3}...x_{n}$ , the reverse sequence ${x^{r}=x}_{n}x_{n-1}...x_{1},$ complement sequence $x^{c}=x_{1}^{c}x_{2}^{c}x_{3}^{c}...x_{n}^{c}$ , and reverse-complement sequence $x^{rc}=x_{n}^{c}x_{n-1}^{c}...x_{1}^{c}$ were used. We applied the c mark to indicate the Watson–Crick complement of DNA nucleotide, thus $T^{c}=A,A^{c}=T,G^{c}=C$ and $C^{c}=G$ (Aboluion et al., 2012). For instance, the DNA sequences AAGGTACT, AGTACCTT, and TGAAGCAT are the reverse, complement, and reverse-complement sequences for the TCATGGAA.

In retrospective studies (Cao et al., 2020b; Rasool et al., 2022a), some sequences were similar among the set of DNA sequences, including reverse or reverse-complement, and then non-specific hybridization occurred, which caused errors by forming the secondary structure (SS). A sequence structure in which a set of pairs of nucleotides support a secondary structure is known as the stem, while the number of repeated nucleotides is known as the stem length. The secondary structure is a result of similar sequences with base-pairing connections in which one sequence folds back to itself (Heckel et al., 2019). DNA sequence with a secondary structure makes chemical reaction less active, which causes the redundancy of that DNA sequence during the synthesis and sequencing process. Thus, the secondary structure must be unfolded before reading such sequences in the wet lab. Constructing robust DNA codes with fewer resources and errors will be helpful. This problem motivated us to put forward a novel solution that must deliver sufficiently different DNA sequences among a set of sequences by evading the secondary structure.

To avoid the SS before DNA reading, one can eliminate the base–pair connection by selecting a sequence with appropriate nucleotides that must have a sufficient code rate, R, and Hamming distance, d . The net code rate $R=\log_{4}k/n$ , where k is the number of codes in a set (coding set) and n is the sequence length number. For example, let us take a set of quinary DNA codewords $\Sigma =\left\{AT,AC,CT,CG,TG\right\}$ with length 2 n . Any DNA code with such length is designed by a bijective map between quinary codes and fixed Σ , then the code rate $\log_{4}5/2$ ≈ 0.5804. Thus, in this paper, we considered the SS with a positive integer m having stem length l and sequence length with n−1 . In this case, Theorem 1 constructs the mathematical proof to design the DNA sequences free from secondary structure.

Theorem 1 *A DNA sequence with length* n *is free from the SS if there does not exist a sequence of the stem length* l>1 *with a minimum Hamming distance* $d_{H}=d$ *and* n−1 *.*

**Proof.** For a given sequence $x=x_{1}x_{2}x_{3}...x_{n}$ , two subsequences $x_{i}x_{i+1}...x_{i+m-1}$ and $x_{j}x_{j+1}...x_{j+m-1}\exists$ $\left|i-j\right|>m$ and ${\left(x_{i}x_{i+1}...x_{i+m-1}\right)}^{sc}=x_{j}x_{j+1}...x_{j+m-1}$ ; then, these two subsequences are known as disjoint secondary-complement. It should be noted that a $x^{rc}$ is a secondary-complement (sc) sequence, but all secondary-complement sequences may not be $x^{rc}$ . In this scenario, if there is a secondary structure with the stem length l>1 , there will be two disjoint subsequences (x and y) ∃ $x=y^{sc}$ . Thus, the results evolve as contrapositive logic for such observation that if a sequence $x=x_{1}x_{2}x_{3}...x_{n}$ is free from SC subsequences with length l and n−1 , then the corresponding DNA sequence will also be freed from the SS.

Apart from the SS problem, the deletion and substitution errors ε and Hamming weight M in the lower bounds caused the density of the DNA data storage system. Proposition 12 (King, 2003) leads us to construct Theorem 2 (**Note 01** in the Supplementary Materials) based on reverse-complement with K-constraint length. The purpose of this theorem is to improve the lower bounds of DNA codes by RC constraints for a maximum number of DNA sequences with minimum errors r . It is constructed by Shannon’s relationship, considering the redundancy of explicit DNA codes with ⌊r/2⌋ log⁡M.

## 3 Evolutionary approach

The proposed evolutionary approach leverages the development of the MFO (Mirjalili, 2015) with DNA coding constraints. The MFO algorithm is selected because it can solve the challenging constraints and unknown search space problems for several applications, i.e., sequence compression problems, which motivated us to generalize this algorithm with DNA coding constraints. Moth belongs to the butterfly family with various similar characteristics. It has a particular navigation mechanism known as transverse orientation (TO) to fly by using the moonlight. The TO mechanism empowers the moth to fly by regulating a fixed angle by the Moon’s focal point, which allows it to fly long distances. Meanwhile, the moth often collides with human-made light due to being closer than moonlight, which distracts the moth from the destination. However, the moth endures in preserving the same angle, which causes its deadly spiral path. Eventually, this behavior supports the convergence of the moth toward the light. This concept provides a mathematical optimizer algorithm termed a moth-flame optimizer (MFO) for object convergence (Mirjalili, 2015).

This evolutionary algorithm has two candidate solutions, moth and flame, and one problem variable is the position of the moth during flight. Thus, a moth can fly by changing the position in 1/2/3 or hyper dimension of space. The moth candidate solution can be considered in the following matrix Q due to its population-based system.

$$Q=\left[\begin{array}{c} \begin{array}{ccc} \begin{array}{c} q_{1,1}\\ q_{2,1} \end{array} & \begin{array}{cc} q_{1,2} & \cdots \\ q_{2,2} & \cdots \end{array} & \begin{array}{cc} q_{1,d-1} & q_{1,d}\\ \cdots & q_{2,d} \end{array}\\ \vdots & \begin{array}{cc} \vdots & \vdots \end{array} & \begin{array}{cc} \vdots & \vdots \end{array}\\ \begin{array}{c} q_{n-1,1}\\ q_{n,1} \end{array} & \begin{array}{cc} \cdots & \cdots \\ q_{n,2} & \cdots \end{array} & \begin{array}{cc} \cdots & q_{n-1,d}\\ q_{n,d-1} & q_{n,d} \end{array} \end{array} \end{array}\right],\;Qf=\left[\begin{array}{c} {Qf}_{1}\\ {Qf}_{2}\\ \begin{array}{c} \vdots \\ {Qf}_{n-1} \end{array}\\ {Qf}_{n} \end{array}\right],$$ (1)

where n indicates the moth numbers and d presents the dimension variable.

An evolutionary population-based algorithm also considers an array of fitness values for all moths. The first row of Q matrix directs the position vector of a moth, which requires passing the fitness function. As a result, a fitness value is assigned to those particular moths as ${Qf}_{1}$ in Qf matrix.

Meanwhile, the second candidate solution of MFO, the flame, also has a similar matrix R as assumed for the flame. The array dimension of both solutions is equal in numbers. Hence, a similar fitness array is also considered for the flame.

$$R=\left[\begin{array}{c} \begin{array}{ccc} \begin{array}{c} r_{1,1}\\ r_{2,1} \end{array} & \begin{array}{cc} r_{1,2} & \cdots \\ r_{2,2} & \cdots \end{array} & \begin{array}{cc} r_{1,d-1} & r_{1,d}\\ \cdots & r_{2,d} \end{array}\\ \vdots & \begin{array}{cc} \vdots & \vdots \end{array} & \begin{array}{cc} \vdots & \vdots \end{array}\\ \begin{array}{c} r_{n-1,1}\\ r_{n,1} \end{array} & \begin{array}{cc} \cdots & \cdots \\ r_{n,2} & \cdots \end{array} & \begin{array}{cc} \cdots & r_{n-1,d}\\ r_{n,d-1} & r_{n,d} \end{array} \end{array} \end{array}\right],\;Rf=\left[\begin{array}{c} {Rf}_{1}\\ {Rf}_{2}\\ \begin{array}{c} \vdots \\ {Rf}_{n-1} \end{array}\\ {Rf}_{n} \end{array}\right],$$ (2)

where n indicates the number of flames and d represents the dimension variable.

It should be noted that moth and flame are both solutions. However, the difference between these solutions is the mechanism we conducted with them to update them in all iterations of processes. The moth’s solution flies in a circular search space by acting as a search agent. In contrast, the flame is the destination solution for the moth in that search space. Hence, the moth struggles to reach the destination as an optimal solution by flying around the search space. Overall, this algorithm (Mirjalili, 2015) mechanism is based on the following three tuples to optimize the problem for an optimal solution.

$$MFO=\left(U,P,T\right),$$ (3)

where U is a function that is responsible for the moth’s random population with its related fitness. This function can be modeled as $U:Ø\to \left\{Q,Qf\right\}$ . Meanwhile, the P function is assumed to fly the moth in a search space. It attains the Q matrix and returns the updated $Q_{i}$ , which is shown as $P:Q\to Q_{i}$ . The third tuple is the T function, which delivers the true value if it satisfies the termination criteria; otherwise, it returns false values; it is shown as $T:Q\to \left\{true,false\right\}$ .

The P function is executed iteratively until it delivers the T function with the true value. The inspiration of this evolutionary algorithm is the TO mechanism by which the moth updates its location by considering the flame location with the following computation model:

$$Q_{i}=S\left(Q_{i},R_{j}\right),$$ (4)

where $Q_{i}$ represents the i -th moth updates, S indicates the spiral function, and $R_{j}$ presents the j -th flame.

**FIGURE 1:**
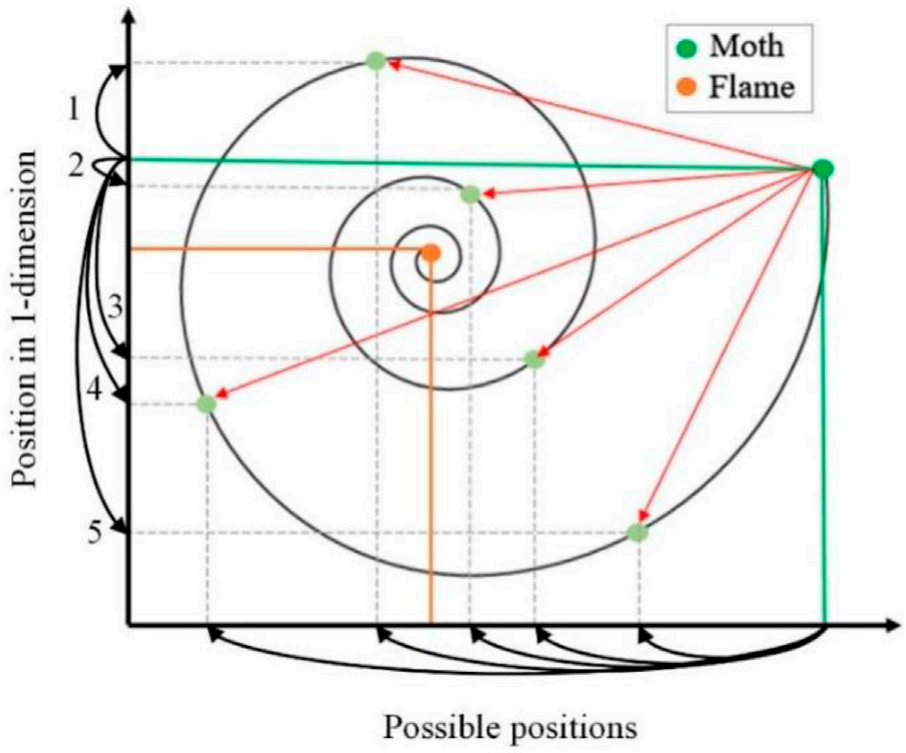
Conceptual model for moth–flame algorithm to update moth’s location according to a flame.

The spiral function is a key component of the MFO, which decides the moth movement with respect to flames. A conceptual model is drawn in Figure 1 for the moth location updates according to the flame. The vertical axis indicates the position of a moth in one dimension, and the horizontal axis presents the possible position update for the next location. The dashed black lines display the locations that can be used for the next position of the moth in the green horizontal line concerning the orange horizontal line of a flame. The exploitation and exploration processes can be determined in the search space. Moth uses one dimension to explore and exploit the search space of flame. Exploitation emerges if the next position between the moth and flame is inside the search space, as directed by arrow 2. In contrast, exploration appears if the position updates between the moth and flame are outside the space, as indicated by arrow 1, 3–5 in Figure 1. Exploration and exploitation can be observed by following three rules.

1) A moth may converge by altering the random number $\left[-1,+1\right]$ in the flame neighbor.
2) The lower random number is the closest distance to the flame.
3) The position frequency increases automatically as the moth comes closer to the flame.

For an effective possible solution, finding the best solution can be considered a flame candidate solution. The Q matrix (1) indicates the best solution gained so far. Apart from these three-tuple-based functions of the MFO algorithm, two consequential parameter-based arrays are also examined as upper and lower bounds:

$$ub=\left[{ub}_{1},{ub}_{2},{ub}_{3},\ldots ,{ub}_{n-1},{ub}_{n}\right],$$ (5)

$$lb=\left[{lb}_{1},{lb}_{2},{lb}_{3},\ldots ,{lb}_{n-1},{lb}_{n}\right],$$ (6)

where the variables ub and lb present the upper and lower bounds with n number of moths, respectively. These variables are responsible for the moth’s local and global search space limit. It must be noted that these considerations are for optimizing the MFO algorithm, while in nature, it might not be a real mechanism.

The evolutionary mechanism of this algorithm permits the moth to attain the best position in the local and global search space. However, a problem can occur that causes the MFO algorithm (Mirjalili, 2015) to fall into the local optima due to only updating the moth position corresponding to the flame and in one dimension. If all moths update the position with respect to only a single flame, all will be converged due to flying toward a flame in the search space that causes the exploration. In contrast, if all moths update the position with only n different locations and dimensions, say 3D dimensions, it stagnates the exploitation. Both cases adversely affect the promising solution of the MFO algorithm. In order to sustain the balance between exploitation and exploration capabilities and find the optimal global solution for the moth, this study employed two mutation strategies to synergy the original evolutionary algorithm: Levy flight and opposition-based learning (OBL) mutation strategy. To determine the optimal global solution, the algorithm compares the output values of the computed global solution $S_{g}$ with the values of global position $P_{g}$ . If the output of $S_{g}>P_{g}$ , the algorithm will return the output $X_{m}$ as the optimal global solution. In this paper, we have omitted the theoretical explanation of the OBL strategy, and readers can see the literature (Rasool et al., 2022a). Meanwhile, the theoretical details of the Levy flight are moved in **Note 02** in the Supplementary Materials.

Meanwhile, the computation time complexity is a crucial metric for evaluating the algorithm run time. It is based on algorithm structure and parameter settings, i.e., number of candidates, location updates, maximum iterations, number of variables, and sorting mechanism. The Quicksort method has been adopted with a time complexity of $O\left(nlogn\right)$ for the best case and $O\left(n^{2}\right)$ for the worst case. Considering the moth move function, P, and the mutation strategies, the overall computation time complexity of the improved MFOS algorithm is as follows:

$$O\left(MFOS\right)=O\left(t\left(O\left(Quicksort\right)+O\left(location\;updates\;with\;mutation\;strategies\right)\right))\right)),$$

$$O\left(MFOS\right)=O\left(t\left(n^{2}+n\times v\right)\right)=O\left(tn^{2}+tnv\right),$$

where t represents the iteration numbers, n indicates the number of moths, and v is the variable number.

In summary, the MFO (Mirjalili, 2015) has the capability to explore and exploit the solution in the search space, while Levy flight enables MFO for the candidate solution to acquire tiny flight with profound exploration and exploitation ability, and the OBL mutation strategy is concerned with the opposite directions for the optimal solution. These strategies significantly improve the MFOS capabilities for jumping from the local area to the promising global areas. The nest subsection investigates the practical implication of constrained optimization with an improved evolutionary approach. The pseudocode of the improved evolutionary algorithm is presented in Algorithm 1. The information about mutation strategies mentioned in Algorithm 1 is provided in **Note 02** in the Supplementary Materials.

Algorithm 1 Pseudocode of the improved MFOS algorithm.

**Input:** The population size N for 2 candidate solutions (Q, R), moth location (L), moth *FitnessFunction* ( $Q_{f}$ ), flame *FitnessFunction* (*R*_*f*_).
**Output:** Optimal global solution ***X***_***m***_
 1: Initialize random population *X*_*i*_;
 2: **for** (each moth *X*_*i*_) **do**
 3: Calculate *Q*_*f*_ and *R*_*f*_ population using Eqs 1, 2;
 4: **if** (population *N* converge), **then**
 5: Update *L* for *lb* & *ub* using Eqs 5, 6;
 6: **else**
 7: Update *Q*_*f*_ and *R*_*f*_ with Eqs 3, 4; **end if end for**
 8: **for** synergy of mutation strategies with Eq. (9) and OBL; **do**
 9: **if** (iteration reaches), **then**
 10: Compute global solution;
 11: **else**
 12: Re-run mutation strategies;
 13: **else if**
 14: Calculate fitness of population; **end else if**
 15: **end if**
 16: **end for**
**Return:** Optimal global solution ***X***_***m***_ **.**

### 3.1 Constrained optimization with MFOS

In order to solve the multi-objective problem of the DNA data storage, the proposed meta-heuristic evolutionary algorithm is applied to the DNA coding constraints (Section 2) to find the lower bounds of DNA code words on coding sets, $C\left(n,W,d\right)$ , where n shows the sequence length, W indicates the GC-content with ⌊n/2⌋ parameters, and d is the Hamming distance. It is convenient to achieve longer codes with repeated values. However, the DNA codes with such sequences are more likely to have errors prone to the nucleotides’ insertion, deletion, or substitution. Therefore, the improved MFOS algorithm tackles this problem by considering computational-based biological coding constraints. Church et al. (2012) reported the influential factors of errors in the sequence and suggested satisfying the GC-content and no-runlength constraints to reduce the sequencing errors (Church et al., 2012). In this regard, this study introduces RC constraints to reduce the error occurrence probability. The DNA code words’ construction that meets the constraint criterion (based on Algorithm 2) is adopted as a primer or address libraries of oligos. These DNA storage code words are more suitable for constructing random storage. Organick et al. (2018) proved that each oligo file can be retrieved without errors from the random-access storage. The development of lower bounds on $C\left(n,W,d\right)$ sets with MFOS achieves the significant number of code words for coding sets that construct the highly robust DNA data storage. The pseudocode for DNA coding constraint’s satisfactory criteria with MFOS is presented in Algorithm 2.

Algorithm 2 Pseudocode of constraints satisfactory criteria with MFOS.

**Input:** Sequence length ( n ), $C^{GC,NL,RC}$ (GC-content, No-runlength, RC constraint, and Hamming distance ( d )), Secondary structure ( SS ).
**Output:** DNA coding sets $C^{GC,NL,RC}$ .
 1: Initialize individual constraint $C_{i}$ ;
 2: **for** (constraints satisfaction) **do**
 3: Update $C^{GC,NL,RC}$ ;
 4: **if** (error occurrence), **then**
 5: remove SS using *Theorem 1*;
 6: **else if** $\left(iter\leq 500\right.$ ) **then**
 7: maximum iteration using *Theorem 2* for best n and d ;
 8: **if** (lower bounds improved), **then**
 9: constraint satisfied;
 10: **else**
 11: update DNA coding constraint; **end if**
 12: **end else if**
 13: $iter=iter+1\;\leftarrow \;C_{n}$ ;
 14: **end if**
 15: **end for**
**Return:** Improved DNA coding sets $C^{GC,NL,RC}\left(n,W,d\right)$ .

**FIGURE 2:**
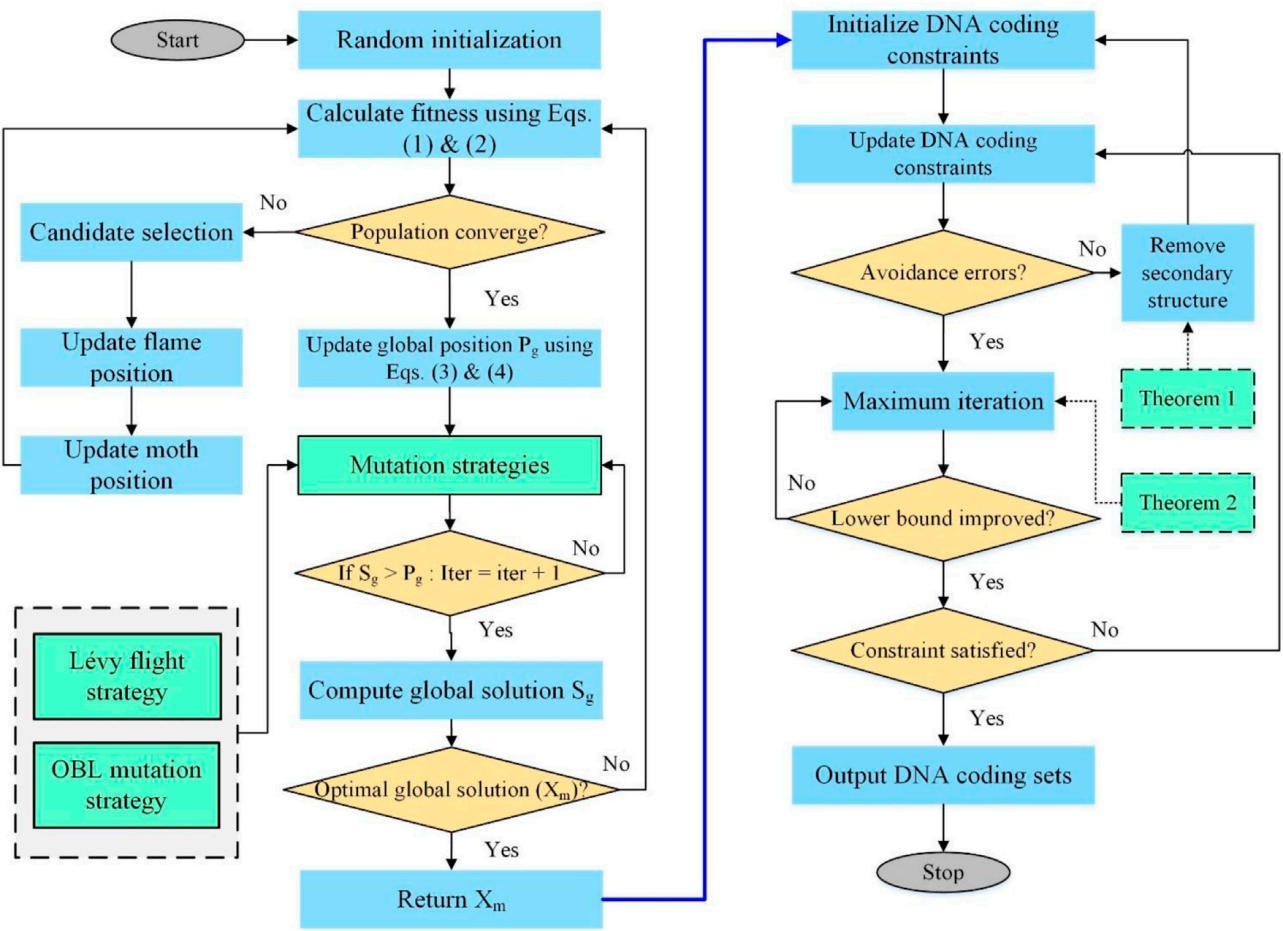
Schematic of the improved MFOS evolutionary approach with combinatorial DNA coding constraints.

The following are MFOS’s fundamental steps to construct DNA storage coding sets with constraints $C^{GC,NL,RC}\left(n,W,d\right)$ , and the schematic of improved MFOS and its implication for DNA storage is illustrated in Figure 2. Concisely, this approach compresses four major components: 1) initialization, fitness, and convergence computation; 2) synergy of mutation strategies for the best global solution; 3) avoidance of errors by theorems; and 4) DNA coding constraints satisfaction.

• The Universe population N is initialized with the best candidate solution from moth and flame, and MFOS algorithm parameters are set with possible DNA coding that follows the particular constraints (GC, No-runlength, and Hamming distance).
• The DNA storage coding set is performed with the initial Universe population, and globally optimal solutions are secured with the current fitness of the candidate solution.
• The lower and upper bounds are computed with Eqs 5, 6 for the candidate solution, updating its exploration and exploitation position with Eq. 3.
• The updated position is executed with two mutation strategies (Levy flight and OBL) to assign the global search space to the candidate for the improved MFOS algorithm.
• The DNA-coding sets with $C^{GC,NL}\left(n,W,d\right)$ constraints are merged with RC constraints ( $C^{GC,NL,RC}\left(n,W,d\right)$ ) to eliminate the non-specific hybridization error using theorems 1 and 2, and the coding set with different iterations is computed.
• If the results of combinatorial constraints satisfy the improved coding rate and find the lower bounds in a higher number of codes, then the new output will be the best DNA data storage coding sets C.

These critical steps have been followed in constructing DNA codes for data storage.

## 4 Experimental setup

The implementation was conducted in an integrated environment of different tools. All the evolutionary algorithms are executed on MacBook 2.4 GHz Intel Q-Core i5, 8 GB 2133 MHz DDR3, language Python with 3.8.11v, Google’s Collaboratory, and 3-dimensional convergence plots into MATLAB R2018b. In the proposed work, an image file (horse.png^1^) is converted into binary data by using the python package (*TransBin*). The binary data are mapped through the quarterly number (A-0, T-1, C-2, and G-3) of DNA bases (A, T, C, and G) for the construction of DNA code words. The proposed MFOS evolutionary algorithm is programmed in Magma software (Cannon et al., 2011) by varying the sequence lengths and Hamming distance. The DNA coding constraint-based theorems are used to generate the least-errors DNA codes with high density and coding ratio. As a result, .cod files are received with different DNA codes that satisfy the GC-content, no-runlength, and RC constraint with different numbers of sequence length and Hamming distance. Consequently, the received DNA codes from .cod files are calculated to construct the lower bound tables. Furthermore, 19 mainstream benchmark functions are implemented with the MFOS algorithm, and the theoretical details are provided in **Note 03** in the Supplementary Materials.

All mainstream functions have been implemented with identical conditions. The number of iterations for each function is set at 500, and the number of moths or population size is adjusted to 50. The MFOS convergence rate directly depends on the population size with crossover and mutation probabilities. The higher the crossover, the greater the exploration ability. Typically, the crossover probability is adjusted in the 0.6–1.0 range. In contrast, mutation probability is often considered lower as compared to crossover, i.e., 0.005 to 0.05 (Wang et al., 2020). It should be noted that the moth number of candidate solutions (i.e., flame) must be selected on an experimental basis. The larger the number of candidates’ solutions, the larger the chances of achieving the global optimum. In this study, the number of moths is reasonably estimated up to 30 times to eliminate the randomness of the optimizer results. However, this number can be 10 or 20 as well for different varieties of experiments. The summary of these operators and their parameter selections is presented in Table 1.

**TABLE 1:** MFOS’s operators and parameter settings.

MFOS operators	Methods and parameters
Population size	50
Number of iterations	500
Parent selection	Random
Population selection	Two best individuals
Crossover probability	0.8
Crossover method	Arithmetic crossover
Mutation probability	0.05
Mutation method	Levy flight and OBL

MFOS is a heuristic algorithm based on the MFO algorithm (Mirjalili, 2015), which must be executed almost 10 times to deliver significant results. It is a general standard that an evolutionary optimizer performing for n times can be evaluated by average (AVG) and standard deviation (SD) with the optimal solution in the last iteration, which will be computed as performance metrics (Mirjalili, 2015). This study used the following rule to report the optimal solution with AVG and SD scores.

• The lowest average score is the highest algorithm performance.
• The minimum standard deviation score is the maximum stability of the algorithm.

In addition, the proposed algorithm MFOS is compared with the original MFO (Mirjalili, 2015) and various well-known and popular optimizer algorithms to validate the performance, for example, the Firefly Algorithm (FFA) (Emary et al., 2015), Grey Wolf Optimizer (GWO) (Mirjalili et al., 2014), Differential Evolution (DE) (Zhang et al., 2011), Multi-Verse Optimizer (MVO) (Mirjalili et al., 2016), and Harris Hawks Optimization (HHO) (Heidari et al., 2019). The FFA is a multi-objective optimizer for various domains and still has multiple variants. GWO achieved suitable compromises between exploration and exploitation. Recently, the MVO algorithm has been popular in DNA coding constraints in various DNA-based storage studies, i.e., DMVO (Cao et al., 2020b). HHO is a swarm-based algorithm that supports multiple iterations. A statistical test is computed from the benchmark functions to ensure the result’s originality. A non-parametric Wilcoxon rank-sum test (Kim and Kim, 1996) is adopted to evaluate and compare the results of MFO and MFOS algorithms.

Furthermore, the MFOS algorithm is applied in the Magma program with DNA coding constraints, i.e., GC-content and RC constraint, to assess the DNA storage effectiveness by the coding sets. The received lower-bound values are compared with a state-of-the-art Altruistic algorithm (Limbachiya et al., 2018) and two recent optimizer algorithms [KMVO (Cao et al., 2020a) and DMVO (Cao et al., 2020b)]. In addition, this study reported the lower bounds of DNA codes with RC constraint and compared the results with previous work, MFOL (Rasool et al., 2022a). A thermodynamic analysis is performed on coding constraints and compared with the newly introduced RC constraint to validate it by the computation of temperature variance.

## 5 Result evaluations

### 5.1 Benchmark function’s evaluation

A general trend exhibits the improved performance of our evolutionary algorithm in many functions. The improved AVG and SD values in the results prove that strategic combination is more effective in gaining convergence for the global optimum than MFO and the other five algorithms. Further evaluations are reported in **Note 04** in the Supplementary Materials.

### 5.2 Convergence efficiency

**FIGURE 3:**
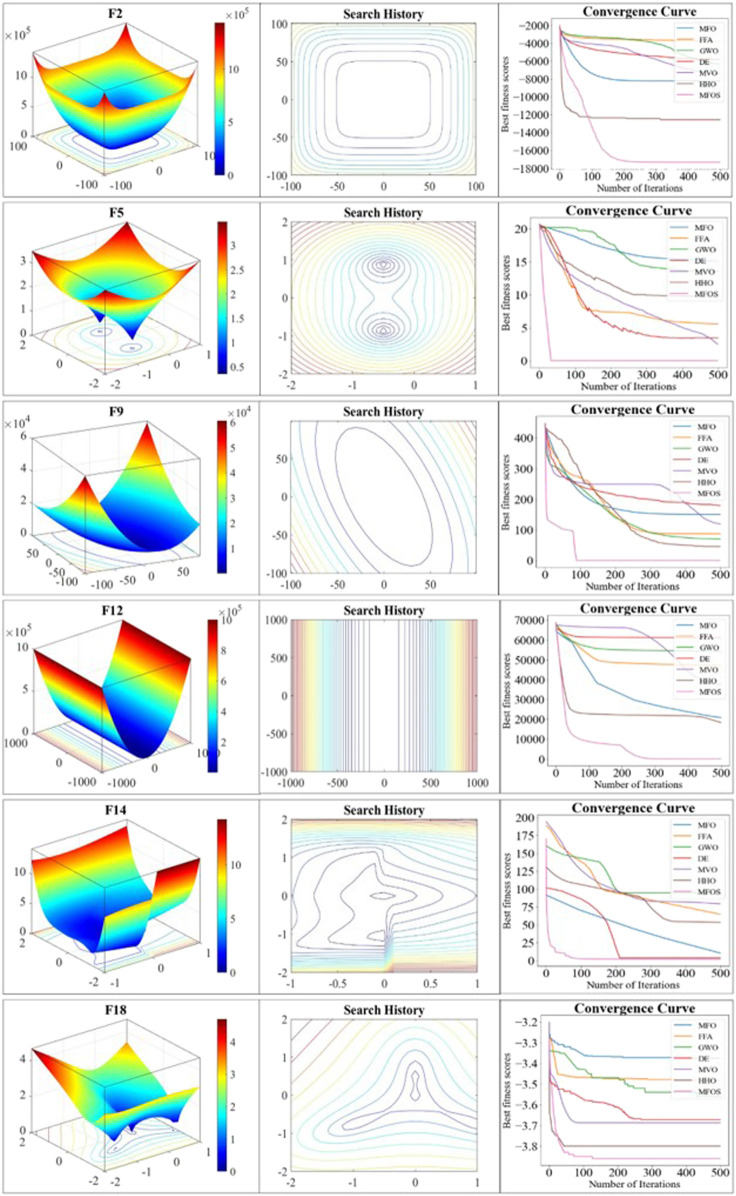
Convergence efficiency of unimodal (F2 and F5), multimodal (F9 and F12), and composite (F14 and F18) functions with 3D search space, search history, and curves.

The convergence curve is a key criterion for evaluating the optimizer’s convergence speed and ability to jump out from the local optima. This study re-executed experiments on the specific mainstream functions by using five moths over 500 iterations. These functions are selected based on the best performance from Tables 1, Table 2, and **3** given in **Note 03** in the Supplementary Materials. Two metrics, search history and convergence rate, are adopted to affirm the convergence of the improved MFOS algorithm. Search history is a qualitative metric that plots the 3D representation curves with the sequence of different iterations. It may also be seen from the 3D representation that fluctuation-flow are greatly dependent on the iteration sequence with different values. These observations guarantee the MFOS’s effectiveness for the optimized problem to attain the balanced transition between exploration and exploitation. In addition, the convergence rate is a quantitative metric that plots the best candidate’s fitness in each iteration. The fitness reduction with the passage of iterations empowers the MFOS convergence. To analyze the effectiveness of the Levy flight and OBL mutation strategies, Figure 3 illustrates the comparison of the original MFO, our MFOS, and the other five algorithms. In the unimodal F2 function, although the MFO secured the optimal global solution, it failed to jump out from the local optima in its early fitness. In contrast, the MFOS algorithm converges more speedily than the MFO and attains the optimal global solution after almost 100 iterations. Meanwhile, our optimizer attained optimal solution at 50 iterations in the F12 function. In contrast, the MFO fell into the local optima. Likewise, in the composite F18 function, the MFO curve tends to be horizontal with no more extended convergence, while the MFOS easily and rapidly converges and accesses the global optima due to its mutation strategies. In summary, the search-based meta-heuristic MFOS convergence curves are empirically insured by quantitative metrics. It is observed that the improved algorithm exhibits competitive results over the state-of-the-art optimizers by endowing a balanced nature between exploration and exploitation.

**TABLE 2:** Superscript identifications and their meanings.

Superscript	Meaning
A	Altruistic algorithm (Limbachiya et al., 2018)
K	KMVO (Cao et al., 2020a)
u	DMVO (Cao et al., 2020b)
L	MFOL (Rasool et al., 2022a)
S	MFOS (our proposed algorithm)

### 5.3 Wilcoxon rank-sum test

Rank-sum statistical tests are used for doubtful distribution; without consideration, either the object is known or unknown. A popular rank-sum test proposed by Wilcoxon is an alternative non-parametric test for ranking two samples (Kim and Kim, 1996). The null hypothesis of the Wilcoxon rank-sum test is typically interpreted as equal medians instead of equal means. The two populations have the same distribution with the same median, which is another way to conceptualize the null. If evidence is found that one distribution is shifted to the left or right of the other, the null hypothesis will be rejected. Rejecting the null proves that the medians of the two populations are different because we assume that our distributions are equal. This study investigates the statistically significant difference between the MFO and MFOS algorithms by computing the rank-sum of any two samples of the 30 iterations.

**FIGURE 4:**
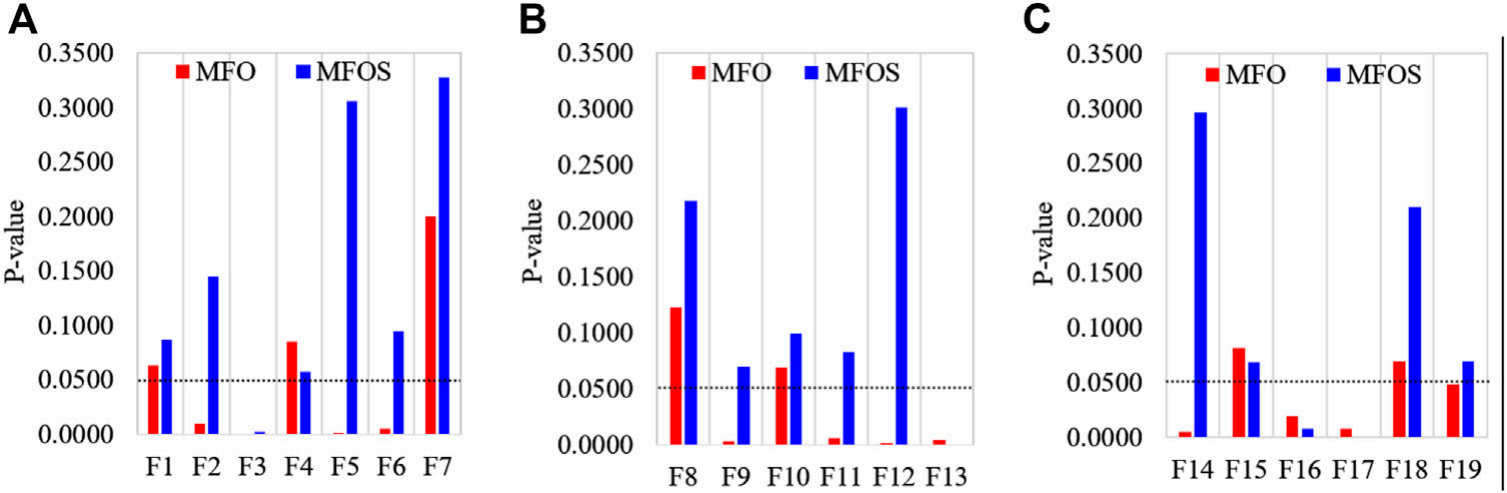
Wilcoxon rank-sum test’s comparison of MFO and MFOS algorithms for **(A)** unimodal functions, **(B)** multimodal functions, and **(C)** composite functions.

Particularly, an algorithm is pondered as statistically substantial if the P−value > 0.05 . In Figures 4A–C, the results met the threshold criteria (*p* > 0.05) in various functions, and the threshold line presents the P−value . These results demonstrate the statistical significance of the MFOS algorithm due to stronger exploration and jumping-out ability from the local optimum.

### 5.4 Bounds on DNA storage coding constraints

The MFOS evolutionary algorithm is programmed to enhance the lower bounds of DNA coding sets with $C^{GC,NL}\left(n,W,d\right)$ constraints which present three GC-content, No-runlength, and Hamming distance constraints that delivered the constraints satisfied DNA sequences. Meanwhile, the acquired MFOS outputs are compared with recently best-obtained results from Altruistic (Limbachiya et al., 2018), KMVO (Cao et al., 2020a), and DMVO (Cao et al., 2020b). The altruistic algorithm (Limbachiya et al., 2018) obtained results by utilizing 4≤n≤13 and 1≤d≤13 constraints with W=⌊n/2⌋ . The algorithms (KMVO (Cao et al., 2020a) and DMVO (Cao et al., 2020b)) found the lower bounds with 4≤n≤10 and 1≤d≤n boundary. The bold entries in Table 3 and Table 4 show the lower bounds of DNA coding sets with our improved MFOS algorithm, and superscript identification is classified in Table 2.

**TABLE 3:** Lower bounds’ comparison of the MFOS algorithm with KMVO (Cao et al., 2020a), DMVO (Cao et al., 2020b), and Altruistic algorithm (Limbachiya et al., 2018) for $C^{GC,NL}\left(n,W,d\right)$ .

n/d	d = 3	d = 4	d = 5	d = 6	d = 7	d = 8	d = 9	d = 10
4	11^a^							
	**12** ^**S**^							
5	20^k,u^	8^k,u^						
	20^**S**^	8^S^						
6	56^k^	24^u^	8^k,u^					
	**63** ^**S**^	**27** ^**S**^	**9** ^**S**^					
7	127^k^	45^k^	17^u^	7^u^				
	**208** ^**S**^	**68** ^**S**^	**23** ^**S**^	**8** ^**S**^				
8	324^u^	106^u^	35^u^	14^u^	5^k,u^			
	**469** ^**S**^	**171** ^**S**^	**42** ^**S**^	**18** ^**S**^	**6** ^**S**^			
9	713^u^	199^a^	65^k^	24^u^	10^k,u^	5^k,u^		
	**1210** ^**S**^	**279** ^**S**^	**90** ^**S**^	**37** ^**S**^	**13** ^**S**^	**6** ^**S**^		
10	2081^k^	555^u^	159^u^	54^k^	20^u^	10^u^	4^a,k,u^	
	**3391** ^**S**^	**829** ^**S**^	**205** ^**S**^	**79** ^**S**^	**25** ^**S**^	10^S^	4^S^	
11	4320^a^	1235^a^	284^a^	82^a^	29^a^	**9** ^**a**^	4^a^	4^a^
	**4703** ^**S**^	**1967** ^**S**^	**429** ^**S**^	**124** ^**S**^	**41** ^**S**^	8^S^	**5** ^**S**^	4^S^
12	**12068** ^**a**^	3326^a^	662^a^	190^a^	58^a^	**22** ^**a**^	8^a^	4^a^
	11967^S^	**5195** ^**S**^	**934** ^**S**^	**509** ^**S**^	**73** ^**S**^	**13** ^**S**^	**9** ^**S**^	**6** ^**S**^
13	41867^a^	7578^a^	1432^a^	1201^a^	123^a^	39^a^	13^a^	6^a^
	**42343** ^**S**^	**8392** ^**S**^	**1780** ^**S**^	**1519** ^**S**^	**197** ^**S**^	**51** ^**S**^	**15** ^**S**^	**9** ^**S**^

**TABLE 4:** Lower bounds of MFOS with $C^{GC,NL,RC}\left(n,W,d\right)$ .

n/d	d = 3	d = 4	d = 5	d = 6	d = 7	d = 8	d = 9
4	11^L^						
	11^S^						
5	24^L^	8^L^					
	21^S^	8^S^					
6	58^L^	26^L^	7^L^				
	**71** ^**S**^	26^S^	**7** ^**S**^				
7	148^L^	49^L^	19^L^	6^L^			
	134^S^	**63** ^**S**^	**23** ^**S**^	5^S^			
8	328^L^	114^L^	35^L^	11^L^	6^L^		
	**419** ^**S**^	**149** ^**S**^	**51** ^**S**^	11^S^	6^S^		
9	906^L^	281^L^	83^L^	30^L^	9^L^	4^L^	
	**1026** ^**S**^	**362** ^**S**^	**113** ^**S**^	**49** ^**S**^	9^S^	4^S^	
10	2254^L^	721^L^	189^L^	79^L^	17^L^	5^L^	5^L^
	2249^S^	**897** ^**S**^	**248** ^**S**^	**127** ^**S**^	**27** ^**S**^	5^S^	5^S^

#### 5.4.1 Lower-bound improvements

New lower bounds reported by the MFOS outperformed other algorithms, shown in Table 3. We analyzed the previous three studies’ lower bounds and then compared our lower bounds with the one with the highest lower bound. For example, at n = 8 and d = 3, the lower bounds of the Altruistic algorithm are 289, KMVO has 319, DMVO obtained 324, and our work achieved 469. Therefore, we compared MFOS with the one with the highest lower bound (DMVO). While few coding sets are on the same previous level, i.e., MFOS has the same sets as KMVO and DMVO when n = 5 and d = 3,4, and n = 11 and d = 10 with an Altruistic algorithm. Similarly, few lower bounds are reported with fewer coding sets as the number of sequence lengths (n) increased. For example, MFOS lower bounds were reduced by 0.83% at n = 12 and d = 3, and 11.11% at n = 11 and d = 8, compared to the Altruistic algorithm. However, with an average of 52.28%, the proposed algorithm improved the lower bound coding sets compared with that of the d = 5. Meanwhile, compared with KMVO and DMVO algorithms in the same state (n = 6–13 and d = 5), the MFOS algorithm’s lower bounds are 12–35% higher than the KMVO and 12–28% higher than the DMVO.

These considerable improvements in MFOS are strengthened by adopting Levy flight and OBL mutation strategies. This strategic method accredits the MFOS algorithm with fast convergence and stronger exploration abilities, enabling it to jump out of the local optima to secure itself in the optimal global solution. In conclusion, the proposed MFOS algorithm considerably acquired a large number of lower-bound DNA-coding sets compared to the benchmark algorithm and designed the conditions to store larger files in the DNA storage system.

Regardless of these substantial developments, the fact that the consecutive repetition of subsequences unaffected with $C^{GC,NL}\left(n,W,d\right)$ is still prone to errors in developing DNA coding sets (Erlich and Zielinski, 2017). Additionally, retrospective studies (Cao et al., 2020b; Rasool et al., 2022a) discovered the SS due to this consecutive repetition of similar subsequences that folds back to themselves. We used the RC constraint with computational modeling to eliminate such an SS to design a stable DNA synthesis and sequencing process. Therefore, this study introduced reverse-complement constraints (Section 2) to construct more robust DNA-coding sets; and the higher the robustness, the lower the error probability in the DNA data storage. RC is added to three basic constraints $C^{GC,NL}\left(n,W,d\right)$ and was implemented with the proposed MFOS algorithm. Table 4 represents the yielded coding sets with 4≤n≤10 and 3≤d≤n bounds that satisfy the four constraints $C^{GC,NL,RC}\left(n,W,d\right)$ . The particular selection of these lower bounds enabled this study to compare the results with MFOL (Rasool et al., 2022a). The results revealed that RC constraints with the MFOS algorithm increased the DNA-coding sets in various n/d. For instance, in all n , 28.12 and 33.52% of DNA coding sets have been improved when d = 4 and d = 5, respectively. In glance of a specific number of sequences, e.g., n = 10 at d = 5, MFOS lower bounds are 31.21% better than the MFOL algorithm.

In contrast, the coding set in a particular Hamming distance is also increased and decreased. For example, in d = 3, 21.13% of lower bounds increased and 11.71% decreased, which is insufficient for DNA data storage construction. It is probably due to the decrement of the MFOS algorithm candidate sets, which decrees the number of appropriate sequences. It indicates there is still room to construct more robust DNA codes without decreasing the lower bounds. For instance, if the candidate sets are increased, n = 10 and d = 7, MFOS can deliver larger sequences with improved lower bounds.

**FIGURE 5:**
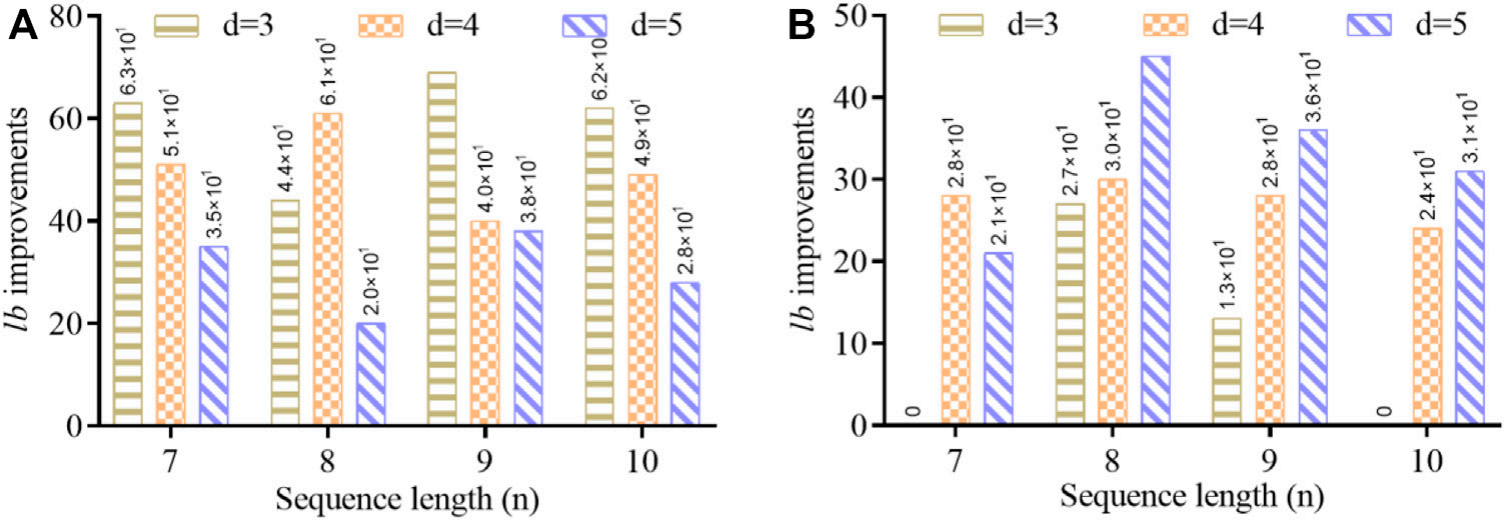
Comparison of improved lower bounds (lb) with different sequence lengths based on **(A)** Table 3 and **(B)** Table 4.

We have illustrated Figure 5 to compare lower-bound improvements for 7≤n≤10 and 3≤d≤5 . Figure 5A is based on the bounds of Table 3, which demonstrates that the GC-content and no-runlength constraints have adequately improved the lower bounds of DNA codes in the lower Hamming distance with all sequence lengths. This improvement is comparatively less with a higher Hamming distance, while it is still more considerable than the existing work with the same constraints. In contrast, Figure 5B reported the lb comparison based on Table 4. It reveals that lower bounds have been significantly improved with a higher Hamming distance with the combination of four constraints, including the RC constraint. The improvement in lower bounds means that the DNA codes given in the particular sequence length have more capacity to accommodate the digital data, improving the density and sequence stability. These consequential performances of the improved algorithm with strategic mutations and the RC constraint confirm its implication for the practical problems of DNA data storage. In addition, the DNA sequences constructed by MFOS for n = 7 and d = 5 are presented in Table 5.

**TABLE 5:** DNA coding sets received at n=7,d=5 from Table 4.

No.	Codes	No.	Codes	No.	Codes	No.	Codes
1	CTGTGAC	7	TAGCCTA	13	GAATGCT	19	CATCGAG
2	ATGTACG	8	TCAGTCA	14	TACGCAG	20	GGCACTA
3	CATCTGC	9	GAGATTC	15	GTACGAT	21	ACGAGTC
4	GCAATCT	10	GTACTAT	16	ACTGACA	22	TGTTACG
5	AGACATG	11	GATGCTA	17	CTACGAT	23	TGAACTG
6	GTCTGAC	12	CTGCCTC	18	GTGACAC	–	–

#### 5.4.2 DNA coding rates

The advancement in the lower bounds is directly favorable to the improvements in DNA coding rates ( R ), which can be defined as (Cao et al., 2020a):

$$R=\log_{4}K/n,$$ (10)

where K indicates the number of DNA-coding sets and n presents the sequence length number.

The empirical analysis of Table 3 revealed that the MFOS algorithm acquired almost the same coding rate with shorter sequence lengths n−1 . For example, the Altruistic algorithm reported *R* = *log*_4_190/12 = 0.315 when n = 12 and d = 6, while the MFOS algorithm reported a 0.316 coding rate when n = 11 and d = 6. Although KMVO and DMVO’s coding rates were better than the Altruistic algorithm, the MFOS’s coding rate was more effective. For example, KMVO informed 0.499 coding rates with n = 7 and d = 3, while the proposed algorithm found 0.498 rates with n = 6 and d = 3. In comparison with DMVO, the MFOS reached 0.323 at n = 7 and d = 5, while DMVO got a 0.32 coding rate at n = 8 and d = 5. Meanwhile, in Table 4, the MFOS algorithm has significantly helped to improve the lower bounds in various coding sets as compared to the previous algorithm (Rasool et al., 2022a). For instance, MFOL achieved a 0.545 coding rate when d = 3 and n = 9, while the current algorithm (MFOS) has attained almost the same coding rate of 0.544 with a shorter sequence $\left(n-1\right)$ at n = 8 with d = 3. Similarly, at d = 5, the MFOS’s coding rate was 0.323 with n = 7. However, the previous MFOL algorithm had 0.32 with n = 8. The evaluation of Table 4 revealed that the improved evolutionary algorithm had increased by 50% in DNA coding sets, while 35.7% of coding sets have the same level as MFOL. These adequate improvements in the lower bounds report the satisfaction of DNA coding constraints.

**FIGURE 6:**
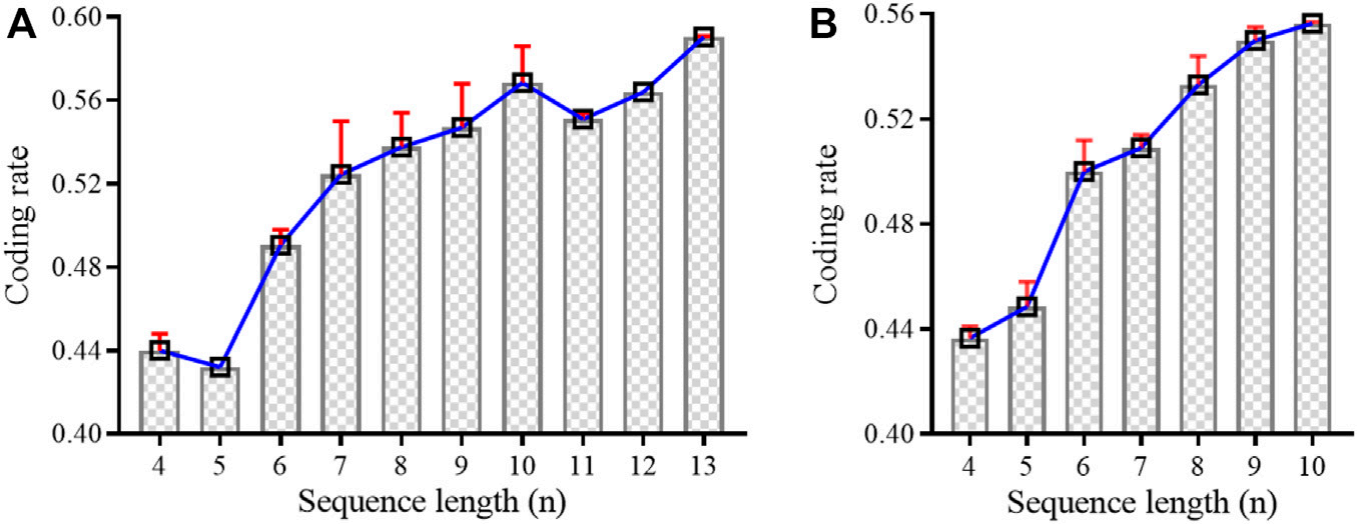
DNA-coding rate comparison at d = 3 using standard error mean based on **(A)** Table 3 and **(B)** Table 4.

Figure 6 compares the DNA-coding rate based on the standard error mean (SEM) for d = 3. The SEM measures how precise the new sample is as the probability of the existing sample. It is used to statistically estimate the interval of the DNA code rate between our new codes with previous codes. Figure 6A presents the coding rate of Table 3 based on $C^{GC,NL}\left(n,W,d\right)$ constraints and provides the SEM between our code rate and the Altruistic algorithm (Limbachiya et al., 2018; Cao et al., 2020a; Cao et al., 2020b). The rising of bars with the increasing sequence length indicates the improving coding rates with n−1 sequence. However, the red error bars specify the estimation of the mean error between our DNA codes and previous ones. It means that the smaller the error bar, the larger the coding rate with the better estimation of DNA codes. Similarly, Figure 6B delivered the same comparison for $C^{GC,NL,RC}\left(n,W,d\right)$ constraints based on Table 4. It emphasizes the effectiveness of combinatorial constraints, wherein four constraints have been configured to attain the maximum coding rate with the slightest mean error. Overall, this evaluation provides the strength that larger digital files can be stored in shorter sequences with n−1 by avoiding the mean error.

Hence, these analytical results determined that shorter sequences can also gain the same DNA storage performance as longer sequences. Consequently, shorter sequences would easily reduce the cost and make it easier to synthesize with more stable conditions. The increased lower bounds in the given coding sets signify the reduction of the SS in the DNA sequences, which helped the proposed approach to avoid the non-specific hybridization errors. These results emphasize the improvement of lower bounds for the further advancement of density-based DNA data storage.

### 5.5 Temperature variance of DNA codes

The temperature variance of DNA codes analytically evaluates the rationality of the newly introduced constraint. In DNA coding, half of the double-stranded DNAs split into single-stranded DNAs during the denaturation process (Sager and Stefanovic, 2006) by the melting temperature ( Tm ). It relies on GC-content that influences the chemical reaction of DNA molecules: the higher the GC-content presents, the higher the Tm . In the PCR, a proper Tm can be more suitable in binding the forward and reverse primers. Hence, the primers with similar Tm can eliminate non-specific hybridization linked with oligo design and its thermodynamic features. DNA sequences with the same Tm are more stable in constructing the DNA codes. This Tm variances are conducted to differentiate the sequence stability: the smaller the temperature variance, the more stable the Tm of the DNA coding set (Chee and Ling, 2008; Dinis et al., 2020).

**FIGURE 7:**
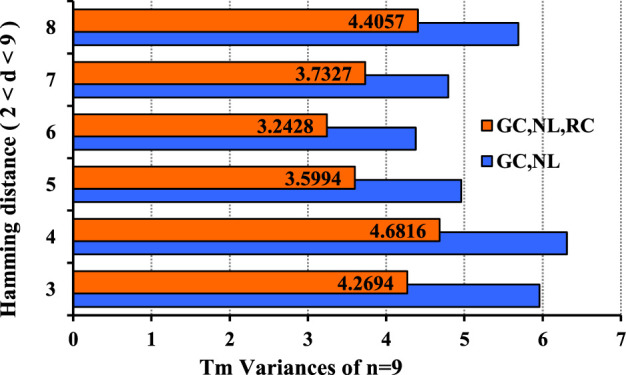
Comparison of Tm variances when n = 9 for the $C^{GC,NL}$ and $C^{GC,NL,RC}\left(n,W,d\right)$ constraints.

As the main objective of this work was to generate a DNA sequence with shorter sequences, an experiential thermodynamic test was performed to authenticate the DNA sequences. The experiential parameters of primer concentration are set at 200nM, and the salt concentration at 50nM . For instance, based on these concentrations, a primer (ACGTATCAGA) with n=10 reported 30% GC-content, and nucleotide degeneration is allowed at Tm=26°C . The coding sets with our evolutionary approach are examined with and without the newly added constraint for its associated Tm values. Table 6 delivers the comparison of Tm variances with $C^{GC,NL}$ ; $C^{GC,NL,RC}$ constraints for particular (6 < n < 10, 2 < d < 9) lower bounds. The results demonstrate the lowest Tm variance for the new constraint ( $C^{GC,NL,RC}$ ) compared to $C^{GC,NL}$ . For comprehensive insights, a clustered bar chart is illustrated in Figure 7. It compares the Tm variances for $C^{GC,NL}$ and $C^{GC,NL,RC}\left(n,W,d\right)$ when n = 9 and Hamming distance is 2 < d < 9. It indicates the 22% smaller Tm variance for the new constraint as compared to $C^{GC,NL}$ . The lowest Tm variances for $C^{GC,NL,RC}\left(n,W,d\right)$ indicates significantly more effective results for the RC than the existing constraints. This analysis dominates the practical inference and demand of the RC constraint for DNA-coding sets as it decreases 13.36–37.99% Tm variances when n = 9. These smaller Tm variances of the DNA-coding set provide support to the stable PCR reaction due to the reduction of non-specific hybridization, which proved the RC constraint applicability.

**TABLE 6:** Comparison of $C^{GC,NL}$ and $C^{GC,NL,RC}\left(n,W,d\right)$ for Tm variance of DNA codes with 5 < n < 10, 2 < d < 9.

n/d		d = 3	d = 4	d = 5	d = 6	d = 7	d = 8
7	C^GC,NL^	5.2569	5.3208	5.5191	4.4609	–	–
	C^GC,NL,RC^	4.0324	5.1284	4.7892	4.7177	–	–
8	C^GC,NL^	4.1352	5.671	5.3907	5.3168	4.0051	–
	C^GC,NL,RC^	5.4981	4.7806	5.1395	5.4902	4.8491	–
9	C^GC,NL^	5.961	6.3107	4.9608	4.3775	4.7926	5.6901
	C^GC,NL,RC^	4.2694	4.6816	3.5994	3.2428	3.7327	4.4057

## 6 Conclusion

Computationally optimized DNA storage algorithms have uncertainty for DNA code stability as required by the DNA synthesis and sequencing process. We, therefore, applied mutation strategies to enhance the optimization efficiency by improving the lower bounds, which allowed us to construct stable DNA codes with the minimum hybridization errors. We synergized a bioengineering-based evolutionary approach with the MFOS algorithm and RC coding constraints to construct more stable and robust codes for DNA storage. The results presented in Figures 3, 4 indicate the MFOS’s competence, faster convergence, and better optimization efficiency than previous algorithms. The improved heuristic algorithm is practiced to generate the optimized DNA code words with the GC-content, Hamming distance, no-runlength constraints, and improved 12–28% lower bounds coding sets than the prior algorithm (DMVO), as revealed in Table 3. In addition, the RC constraint with MFOS meaningfully enhanced the coding rate in 50% of the lower bounds at the same constraints compared with the existing work (Table 4). It attained effective coding rates with shorter sequences by avoiding the SS and enhancing DNA storage density and stability. Consequently, our approach’s shorter sequences can help reduce errors during DNA synthesis and sequencing. Thus, storing a larger file in a shorter DNA sequence is feasible due to the improved lower bounds and coding rates of DNA coding sets. Eventually, comparing temperature variances ( Tm ) with and without the RC constraint demonstrates that smaller Tm variances of DNA sequences can avoid further non-specific hybridization for stable DNA data storage. This evolutionary approach enhances the robustness of optimization algorithms for a biological purpose: the construction of DNA coding sets for the future digital data storage technology into DNA. The proposed approach will apply to constructing sequence data in other bioengineering technologies.

In the future, the DNA codes generated by the MFOS algorithm can be assessed under more strict constraints, i.e., hairpin structure or triplet-based unpaired constraints (Zhu et al., 2021). As the constraints are strict, the DNA-coding set will be smaller and have a higher density of DNA data storage codes. Similarly, various lower bounds and coding rates are still captivating to be enhanced for more stable DNA codes. Additionally, the constructed coding sets by the proposed approach can be used to design an end-to-end DNA data storage system (Takahashi et al., 2019).

## Data Availability

The datasets presented in this study can be found in online repositories. The names of the repository/repositories and accession number(s) can be found in the article/Supplementary Material.

## Footnotes

1
http://clipart-library.com/clipart/horse-clip-art-9.htm
