# Supplementary material for: Evolutionary approach to construct robust codes for DNA-based data storage
Source: Front Genet. 2023 Mar 20;14:1158337. doi: 10.3389/fgene.2023.1158337 (PMC10067891; doi:10.3389/fgene.2023.1158337)
Supplement: Supplementary file 1 [file Table1.DOCX]

Supplementary Material

# Note 01

**Theorem 2.** *If* $M, K, r,$ *and* $\varepsilon$ *are positive integers with* $r$*and fixed* $\varepsilon$*, then* $K>3logM+ \varepsilon$*, and the redundancy of improved lower bounds will be* $\left\lfloor\frac{r}{2} \right\rfloor log M+\left\lfloor\frac{r}{2} \right\rfloor-O\left( 1 \right).$

**Proof.** We consider a sequence $x=x_{1}x_{2}x_{3}...x_{M}$ in descending lexicographic order with discrete code, for which each sequence length $K_{\varepsilon}$ follow $2^{\varepsilon}$times errors. Thus, the positive equivalence integer classes $m$ with Hamming weight $M$ run $2K_{\varepsilon}$times. This class m is known as (see p. 360 in [1])

$$m= \sum_{j=0}^{2^{K}} {(-1)}^{j} \binom{2^{K}}{j}\binom{2^{K}+M-j\left( 2+1 \right)-1}{2^{K}}$$

We allocate a lower bound on $m$ by adjusting the bounds for $1\leq i\leq m_{1}$, where $m_{1}\leq m$ and $m_{1}<i\leq m$ with weight $M$ of discrete codes,

$\boldsymbol{m}_{\mathbf{1}}\mathbf{=}\binom{\mathbf{2}^{\boldsymbol{K}}}{\boldsymbol{M}}\boldsymbol{\leq}\frac{\binom{\mathbf{2}^{\boldsymbol{K}}}{\boldsymbol{M}}}{\mathbf{2}^{\boldsymbol{M}}}$. (1)

The expression $\binom{2^{K}}{K}$ provides multiple options for discrete codes, and $K^{M-K}$ counts the remaining $M-K$ sequences as repetitions of the K discrete ones, hence,

$m-m_{1}=\sum_{K=1}^{M-1} \binom{2^{K}}{K}K^{M-k}\leq\binom{2^{K}}{M-1}M^{2}$ (2)

Equation (1) is larger than (2) w.r.t. discrete codes in each given sequence k:

$m_{1}/m-m_{1}= \frac{\left( 2^{K}-M+1 \right)}{M^{3}} \geq2^{K-1-3 \log M}\geq1.$ (3)

Hence,

$m\leq\frac{\binom{2^{K}}{M}}{2^{M-1}}$ (4)

Now, suppose $Š$ is an error-correcting code, then according to the Pigeonhole rule, the size of the sequence is the least $Š/m$ for a particular class $X$, which denotes $X\triangleqŠ$. Thus,

$\left| X \right|\geq\frac{|Š|}{m}\geq\frac{|Š|}{{\binom{2^{K}}{M}}/{2^{M-1}}}$ (5)

We supposed that the DNA codes with improved lower bounds have minimum Hamming distance $d$; in contrast, if two DNA codes have Hamming distance $d+1$, then these corresponding codes in class $Ȿ$ could be unconstructive. Hence, the length of deletion and substitution errors can be controlled by Hamming distance $d$ in the same class $|X|$,

$|X|\leq\frac{2^{M}}{\sum_{i=0}^{\left\lfloor\frac{r}{2} \right\rfloor} \binom{M}{i}{(2-1)}^{i}}$. (6)

By merging (5) and (6), we have that

$|Š|\leq\frac{2\binom{2^{K}}{M}}{\sum_{i=0}^{\left\lfloor\frac{r}{2} \right\rfloor} \binom{M}{i}{(2-1)}^{i}}$ (7)

Hence,

$$\log\binom{2^{K}}{M}-\log\left| Š \right|\geq\log\left( \sum_{i=0}^{\left\lfloor\frac{r}{2} \right\rfloor} \binom{M}{i}\left( 2-1 \right)^{i} \right)-1$$

$=\left\lfloor\frac{r}{2} \right\rfloor log M+\left\lfloor\frac{r}{2} \right\rfloor-O\left( 1 \right)$.

The exponential improvements in lower bounds with the least errors are near twice the lower bounds with GC-content and no-runlength constraints. This paper uses the combinatorial constraints for DNA storage codes more economically by integrating the RC constraint.

# Note 02

## Levy flight mutation strategy

Levy flight is a random walk with powerful disturbance ability, which behaves as an alternative exploration. It deals with short-distance high-frequency exploration and long-distance low-frequency exploration. Many stochastic algorithms have been successfully applied by employing the levy flight strategy and securing sufficient outputs [2]. Its random walk feature is based on the step size, which can be computed by a probability function of levy distribution (power-law tail):

$L \left( X_{i} \right)\approx\left| X_{k} \right|^{1-\alpha} ,$ (7)

where $X_{i}$ indicates the flight length and power-law exponent is restricted with $1 < \leq2$.

The levy stability process with density probability can be defined as [2]:

$f_{L}\left( X; \alpha, \beta\right)= \frac{1}{\pi} \int_{0}^{\infty} \exp\left( -\beta P^{\alpha} \right)\cos\left( PX \right)dP ,$

where $\alpha$ presents the distribution index while $\beta$ chooses the scale properties and unit.

The integral defines the analytical solution, which needs the expansion method if the $X$ achieves the maximum value as follows:

$f_{L}\left( X; \alpha, \beta\right) \approx\frac{\beta\gamma\left( 1+ \alpha\right)\sin\left( \frac{\pi\alpha}{2} \right)}{\pi X^{\left( 1+ \alpha\right)}} ,$

where $\gamma$ denotes the Gamma function with integer $\alpha$ numbers, $\gamma\left( 1+ \alpha\right)$ is equal to $\alpha$.

Further, Mantegna et al. (1994) reported a fast and authentic algorithm for the levy flight mutation with an index distribution $\alpha$ value (0.30–1.99) [2]. In this study, the Mantegna method is employed with levy flight distribution (8) as follows:

$Levy \left( L \right)=0.05 \times\frac{X}{{|X_{k}|}^{1/\alpha}} ,$ (8)

where $X$ and $X_{k}$ are two normal distribution parameters with a standard deviation of $\rho X$ and $\rho X_{k}$ as follows:

$X= \rho( 0, \omega_{x}^{2})$,

$X_{k}= \rho\left( 0, \omega_{k}^{2} \right),$

where $\rho$ is normal distribution, while $\omega$ as follows:

$\omega= \left[ \frac{\gamma\left( 1+ \alpha\right)\sin(\frac{\pi\alpha}{2})}{\gamma\left( \frac{1+ \alpha}{2} \right)\alpha2^{\frac{(\alpha-1)}{2}}} \right]^{1/\alpha}.$

The levy flight strategy (9) is adopted for the MFOS algorithm to update the location of $n$ moth with corresponding flames to attain the best global solution $X_{m}$ as follows:

$X_{m}=X_{i}\times Levy\left( L \right), i \in\left\{ 1,\ldots,N \right\},$ (9)

where $Levy\left( L \right)$ indicates the levy flight distribution with the random numbers to attain the global best solution.

Levy flight is often related to tiny steps and rarely to long jumps, which enable it to search deeply and efficiently in the neighborhood with tiny steps and far areas with long steps. This strategy permits the proposed algorithm to search in the space over a long distance to find the optimal solution. However, due to its tiny steps, it merely covers the whole area in one domain. Therefore, this study adopted the opposition-based learning strategy to cover all possible areas in the opposite direction of a domain for the effective explorer-exploiter framework.

# Note 03

## Mainstream Functions

In literature [3], various mathematical benchmark functions have been implemented to assess the MFO algorithm's effectiveness. This study also employed those 19 mainstream functions to demonstrate the efficiency of MFOS. The key purpose is to test the performance and superiority of the proposed optimizer algorithm over the original MFO [3] and other existing algorithms. These benchmark functions are fundamentally divided into the following three categories.

- **Unimodal Functions (F1-F7):** These functions engage only with 1 global optimal score and do not consider the local optimal score. These functions have the exploitation capability to assess the optimizer performance in the search space.
- **Multimodal Functions (F8-F13):** In contrast to unimodal, these functions have exploration capability due to having numerous numbers of locally optimal solutions, which increase with the number of dimensions. It enables access to the global optimum by leaving the local optimum space.
- **Composite Functions (F14-F19):** Likewise, composite functions have 1 optimal global solution and numerous local optimal values. This feature augments the optimal solution, and the algorithm gains the ability to jump out of the local optimal for an effective search.

The mathematical equations of these functions have been presented in Tables 2, 3, and 4, respectively. The parameter range is the search space boundary, while $Dim$ indicates the function dimension and $f_{min}$ is the optimal global solution. These 19 functions are thumb rule functions for the optimization problems to test the algorithm optimization performance. In this paper, the constraint values are adjusted in the range parameters to achieve reliable test results. Generally, all problems can't be solved by only one algorithm, and all test functions cannot achieve the required results. Thus, the proposed MFOS algorithm can't yield the optimal solution on all benchmark functions.

# Note 04

The followings are the evaluation of three different categories of benchmark or mainstream functions for the exploitation and exploration ability of different optimizers.

## 4.1 Unimodal functions (F1—F7)

Unimodal functions are liable to evaluate the exploitation ability due to 1 global and 0 local optimal solutions, and results are demonstrated in **Table 2** with the AVG and SD matrices. A general trend exhibits the improved performance of our improved evolutionary algorithm in many functions. For example, the functions F2, F3, F5, and F6 received the best convergence in AVG and SD matrices. Notably, the variances of those functions are also 0, which signifies the data stability for DNA storage. In contrast with the MFO algorithm [3], the AVG score of F1 is reduced by more than 6 times. Meanwhile, the results prove that strategic combination is more effective in gaining convergence for the global optimum as compared to MFO. However, the results of MFOS with F4 lag behind the MFO because of larger optimization intervals. In contrast with the remaining 5 algorithms, our algorithm combatively jumps out of the local optimum and protects itself in the global optimum solution with the least magnitude and variances.

**Table 2**. Comparison of different algorithms' performances with MFOS with average and standard deviation values of unimodal functions.

| **Functions** | **Metrics** | **MFO [3]** | **FFA [4]** | **GWO [5]** | **DE [6]** | **MVO [7]** | **HHO [8]** | **Proposed MFOS** |
| --- | --- | --- | --- | --- | --- | --- | --- | --- |
| F1 | AVG | 8.63E+03 | 3.61E+03 | 6.29E+02 | 3.57E+03 | 3.74E+03 | 1.51E+04 | **1.06E+02** |
|  | SD | 1.48E+04 | 9.78E+03 | 4.80E+03 | 1.08E+04 | 8.31E+03 | 2.14E+04 | 1.26E+03 |
| F2 | AVG | **-7.61E+03** | 3.55E+03 | 4.18E+03 | **-5.13E+03** | **-5.37E+03** | **-1.22E+04** | **0.00E+00** |
|  | SD | 1.26E+03 | 2.22E+02 | **1.91E-02** | 6.77E+02 | 1.34E+03 | 1.02E+03 | **0.00E+00** |
| F3 | AVG | 3.50E+04 | 1.75E+04 | 3.24E+03 | 3.77E+04 | 1.30E+04 | 4.77E+04 | **0.00E+00** |
|  | SD | 1.65E+04 | 2.52E+04 | 1.28E+04 | 1.78E+04 | 1.76E+04 | 3.53E+04 | **0.00E+00** |
| F4 | AVG | 6.44E+00 | **1.38E+01** | **3.51E+00** | 4.31E+01 | 2.36E+01 | 5.62E+01 | 2.18E+01 |
|  | SD | 6.55E+00 | 2.26E+01 | 1.29E+01 | 2.50E+01 | 1.97E+01 | 9.36E+00 | 1.06E+03 |
| F5 | AVG | 2.01E+07 | 7.58E+06 | 1.63E+06 | 8.61E+06 | 6.03E+06 | 9.15E+07 | **0.00E+00** |
|  | SD | 4.94E+07 | 2.76E+07 | 1.60E+07 | 3.38E+07 | 2.56E+07 | 1.04E+08 | **0.00E+00** |
| F6 | AVG | **3.44E+01** | 5.04E+04 | 5.59E+04 | 6.15E+04 | 5.78E+04 | 2.36E+04 | **0.00E+00** |
|  | SD | **1.26E+04** | 5.10E+03 | 2.64E+03 | 8.52E+02 | 1.08E+04 | 6.78E+03 | **0.00E+00** |
| F7 | AVG | **1.55E+01** | 4.75E+00 | **7.31E-01** | 4.14E+00 | 2.98E+00 | 3.57E+01 | **2.82E-01** |
|  | SD | 2.04E+01 | **1.41E+01** | 7.04E+00 | 1.58E+01 | **9.23E-01** | 4.50E+01 | **2.25E-01** |

## 4.2 Multimodal functions (F8—F13)

The exploration capability for the MFOS has been experimented 30 times independently with multimodal function, and the results are presented in **Table 3**. These functions have a large local optimal solution that increases mountingly with the problem dimension and enables the exploration ability to be more effective. MFOS contributes higher performance with the least AVG and SD as compared to MFO [3]. The AVG and SD values of F8-F10 and F12 acquired the optimal global solution after 500 iterations, signifying the MFOS's jumping-out performance from the local optimum. The magnitude and variance scores of F8 and F9 are zero, and the variance of F12 is also zero as compared to MFO in both matrices. These consequential results demonstrate the demand and significance of strategic learnings (levy flight and OBL) for the improved evolutionary algorithm (MFOS). In addition, our algorithm was ineffective in gaining the maximum global optimal solution for F11. This inadequate performance may be revealed due to the moth's large interval for optimization in search space.

**Table 3.** Comparison of different algorithms' performances with MFOS with average and standard deviation values of multimodal functions.

| **Functions** | **Metrics** | **MFO [3]** | **FFA [4]** | **GWO [5]** | **DE [6]** | **MVO [7]** | **HHO [8]** | **Proposed MFOS** |
| --- | --- | --- | --- | --- | --- | --- | --- | --- |
| F8 | AVG | **1.09E+04** | 3.33E+10 | 2.81E+10 | 1.79E+09 | 1.75E+10 | 8.32E+09 | **0.00E+00** |
|  | SD | 1.18E+11 | 7.43E+11 | 6.28E+11 | 3.17E+10 | 3.13E+11 | 1.82E+11 | **0.00E+00** |
| F9 | AVG | 1.92E+02 | **1.64E+02** | 1.50E+02 | 2.21E+02 | 2.32E+02 | **1.60E-02** | **0.00E+00** |
|  | SD | 6.75E+01 | 9.48E+01 | **8.45E-01** | 4.65E+01 | 5.14E+01 | 1.26E+02 | **0.00E+00** |
| F10 | AVG | 1.69E+01 | 8.56E+00 | 1.65E+01 | 7.17E+00 | 9.06E+00 | **1.19E+01** | **4.95E-01** |
|  | SD | **1.70E+00** | 3.99E+00 | **2.75E+00** | **5.24E+00** | **4.26E+00** | **2.88E+00** | **5.65E-01** |
| F11 | AVG | 9.18E+01 | **1.12E+00** | **5.71E+00** | 3.13E+01 | 3.53E+01 | 1.40E+02 | 1.74E+00 |
|  | SD | **1.34E+02** | 8.87E+01 | 4.35E+01 | **1.50E+00** | **7.60E-01** | 2.02E+02 | 2.89E+01 |
| F12 | AVG | 3.50E+07 | 1.34E+07 | 3.43E+06 | 1.78E+07 | 1.13E+07 | 2.57E+08 | **1.57E+00** |
|  | SD | 1.01E+08 | 5.70E+07 | 3.56E+07 | 7.82E+07 | 5.82E+07 | 2.76E+08 | **1.26E+00** |
| F13 | AVG | 1.00E+08 | 2.88E+07 | 7.02E+06 | 3.45E+07 | 6.69E+07 | 4.23E+08 | **2.70E-03** |
|  | SD | 1.98E+08 | 1.16E+08 | 7.09E+07 | 1.40E+08 | **1.69E+01** | 4.89E+08 | **5.45E-05** |

## 4.3 Composite functions (F14—F19)

The composite functions are challenging for the search space due to the combination of rotated and shifted multimodal functions. However, those functions are almost identical to the natural search space for balancing the exploration and exploitation capability of the meta-heuristic algorithm. **Table 4** illustrates the performance of the composite functions with the improved MFOS with two matrices (AVG and SD). The AVG and SD scores of the F14, F18, and F19 test functions outperformed the other functions with different magnitudes and variances. However, the MFOS does not deliver effective results for F15, F16, and F17, although these functions successfully reach the global optimum solution for the given iterations. Notably, accurate estimation for global optima is difficult in composite functions, while the results presented the balanced ability of MFOS for exploration and exploitation. Meanwhile, in contrast with the remaining optimizers, the improved algorithm still has sufficient and capable effects to prevent itself from falling into the local optima. Eventually, compared with unimodal and multimodal benchmark functions, the composite functions' performance is merely due to its complex search space.

**Table 4.** Comparison of different algorithms' performances with MFOS with average and standard deviation values of composite functions.

| **Functions** | **Metrics** | **MFO [3]** | **FFA [4]** | **GWO [5]** | **DE [6]** | **MVO [7]** | **HHO [8]** | **Proposed MFOS** |
| --- | --- | --- | --- | --- | --- | --- | --- | --- |
| F14 | AVG | 4.68E+01 | 1.02E+02 | 1.13E+02 | 3.41E+01 | 1.04E+02 | 8.37E+01 | **1.50E+00** |
|  | SD | 2.31E+01 | 3.37E+01 | 2.39E+01 | 3.92E+01 | 3.14E+01 | 2.42E+01 | **1.05E+01** |
| F15 | AVG | **9.80E-04** | 2.26E-03 | 1.07E-02 | **1.10E-03** | 3.04E-03 | 1.04E-03 | **1.42E-03** |
|  | SD | 7.63E-03 | **1.92E-02** | 1.02E-02 | 7.31E-03 | 1.66E-02 | **1.49E-02** | **1.30E-02** |
| F16 | AVG | **-1.02E+00** | **-1.02E+00** | **-1.02E+00** | 1.02E+00 | 1.01E+00 | **1.03E+00** | **-1.02E+00** |
|  | SD | 7.51E-02 | 7.75E+02 | 9.19E-02 | 8.37E-02 | **1.87E-01** | 3.56E+02 | 1.23E+01 |
| F17 | AVG | 4.16E-01 | 4.24E+01 | 4.17E-01 | **4.14E-01** | 4.23E-01 | 4.18E-01 | **4.51E-01** |
|  | SD | 1.45E-01 | 1.51E+01 | **1.52E-01** | 8.70E-02 | **1.58E-01** | **1.35E-01** | **2.04E-01** |
| F18 | AVG | 3.29E+00 | **3.58E+00** | 3.23E+04 | 3.27E+00 | 8.82E+00 | 1.23E+01 | **-3.05E+00** |
|  | SD | **2.74E+00** | 4.87E+00 | 3.64E+00 | 2.28E+00 | 2.79E+00 | 3.10E+00 | **2.09E-01** |
| F19 | AVG | **-3.86E+00** | **-3.85E+00** | **-3.85E+00** | **-3.84E+00** | **-3.84E+00** | **-3.82E+00** | -3.82E+00 |
|  | SD | 3.40E-02 | 4.07E-02 | 3.68E-02 | 6.42E-02 | 6.42E-02 | 4.24E-02 | 2.58E-02 |

# References

[1] C. A. Charalambides, *Enumerative Combinatorics, CRC Press Series on Discrete Mathematics and Its Applications; Chapman & Hall/CRC: Boca Raton, FL, USA, 2002.*

[2] R. N. Mantegna, "Fast, accurate algorithm for numerical simulation of Lévy stable stochastic processes," (in eng), *Phys Rev E Stat Phys Plasmas Fluids Relat Interdiscip Topics,* vol. 49, no. 5, pp. 4677-4683, May 1994, doi: 10.1103/physreve.49.4677.

[3] S. Mirjalili, "Moth-flame optimization algorithm: A novel nature-inspired heuristic paradigm," *Knowledge-Based Systems,* vol. 89, pp. 228-249, Nov 2015, doi: 10.1016/j.knosys.2015.07.006.

[4] E. Emary, H. M. Zawbaa, K. K. A. Ghany, A. E. Hassanien, and B. Parv, "Firefly Optimization Algorithm for Feature Selection," presented at the Proceedings of the 7th Balkan Conference on Informatics Conference, Craiova, Romania, 2015. [Online]. Available: <https://doi.org/10.1145/2801081.2801091>.

[5] S. Mirjalili, S. M. Mirjalili, and A. Lewis, "Grey Wolf Optimizer," *Advances in Engineering Software,* vol. 69, pp. 46-61, 2014/03/01/ 2014, doi: <https://doi.org/10.1016/j.advengsoft.2013.12.007>.

[6] L. Zhang, X. Xu, C. Zhou, M. Ma, and Z. Yu, "An Improved Differential Evolution Algorithm for Optimization Problems," in *Advances in Computer Science, Intelligent System and Environment*, Berlin, Heidelberg, D. Jin and S. Lin, Eds., 2011// 2011: Springer Berlin Heidelberg, pp. 233-238.

[7] S. Mirjalili, S. M. Mirjalili, and A. Hatamlou, "Multi-Verse Optimizer: a nature-inspired algorithm for global optimization," *Neural Computing and Applications,* vol. 27, no. 2, pp. 495-513, 2016/02/01 2016, doi: 10.1007/s00521-015-1870-7.

[8] A. A. Heidari, S. Mirjalili, H. Faris, I. Aljarah, M. Mafarja, and H. Chen, "Harris hawks optimization: Algorithm and applications," *Future Generation Computer Systems,* vol. 97, pp. 849-872, 2019/08/01/ 2019, doi: <https://doi.org/10.1016/j.future.2019.02.028>.
